# Improving the Primary Care Consultation for Diabetes and Depression Through Digital Medical Interview Assistant Systems: Narrative Review

**DOI:** 10.2196/18109

**Published:** 2020-08-28

**Authors:** Geronimo Jimenez, Shilpa Tyagi, Tarig Osman, Pier Spinazze, Rianne van der Kleij, Niels H Chavannes, Josip Car

**Affiliations:** 1 Centre for Population Health Sciences Lee Kong Chian School of Medicine Nanyang Technological University Singapore; 2 Department of Public Health and Primary Care Leiden University Medical Center Leiden Netherlands; 3 Saw Swee Hock School of Public Health National University of Singapore Singapore

**Keywords:** digital medical interview assistant, computer-assisted history taking, primary care, chronic conditions

## Abstract

**Background:**

Digital medical interview assistant (DMIA) systems, also known as computer-assisted history taking (CAHT) systems, have the potential to improve the quality of care and the medical consultation by exploring more patient-related aspects without time constraints and, therefore, acquiring more and better-quality information prior to the face-to-face consultation. The consultation in primary care is the broadest in terms of the amount of topics to be covered and, at the same time, the shortest in terms of time spent with the patient.

**Objective:**

Our aim is to explore how DMIA systems may be used specifically in the context of primary care, to improve the consultations for diabetes and depression, as exemplars of chronic conditions.

**Methods:**

A narrative review was conducted focusing on (1) the characteristics of the primary care consultation in general, and for diabetes and depression specifically, and (2) the impact of DMIA and CAHT systems on the medical consultation. Through thematic analysis, we identified the characteristics of the primary care consultation that a DMIA system would be able to improve. Based on the identified primary care consultation tasks and the potential benefits of DMIA systems, we developed a sample questionnaire for diabetes and depression to illustrate how such a system may work.

**Results:**

A DMIA system, prior to the first consultation, could aid in the essential primary care tasks of case finding and screening, diagnosing, and, if needed, timely referral to specialists or urgent care. Similarly, for follow-up consultations, this system could aid with the control and monitoring of these conditions, help check for additional health issues, and update the primary care provider about visits to other providers or further testing. Successfully implementing a DMIA system for these tasks would improve the quality of the data obtained, which means earlier diagnosis and treatment. Such a system would improve the use of face-to-face consultation time, thereby streamlining the interaction and allowing the focus to be the patient's needs, which ultimately would lead to better health outcomes and patient satisfaction. However, for such a system to be successfully incorporated, there are important considerations to be taken into account, such as the language to be used and the challenges for implementing eHealth innovations in primary care and health care in general.

**Conclusions:**

Given the benefits explored here, we foresee that DMIA systems could have an important impact in the primary care consultation for diabetes and depression and, potentially, for other chronic conditions. Earlier case finding and a more accurate diagnosis, due to more and better-quality data, paired with improved monitoring of disease progress should improve the quality of care and keep the management of chronic conditions at the primary care level. A somewhat simple, easily scalable technology could go a long way to improve the health of the millions of people affected with chronic conditions, especially if working in conjunction with already-established health technologies such as electronic medical records and clinical decision support systems.

## Introduction

Digital medical interview assistant (DMIA) systems, also known as computer-assisted history taking (CAHT) systems, are software programs that allow patients to provide their medical history electronically prior to the consultation, which can be done remotely, via a web-based portal, or on-site in clinic, via tablets or kiosks, before clinical review [1]. DMIA systems have the potential to improve the quality of care and the medical consultation by exploring more patient-related aspects without time constraints and, therefore, acquiring more and better-quality information [2,3].

Primary care is often considered a cornerstone of health care systems [4]. The stronger the country’s primary care orientation, the better the health outcomes it obtains (eg, in terms of all-cause mortality, all-cause premature mortality, and cause-specific premature mortality for several conditions), regardless of the differences in health system characteristics, such as gross domestic product per capita, total physicians per 1000 population members, and percentage of elderly people [5]. As such, one of primary care’s core functions involves diagnosing and treating chronic conditions [6]. Diabetes and depression, two very prevalent chronic conditions, form part of the majority of nonreferred ambulatory visits to office-based physicians [6,7]. The global prevalence of diabetes reached 8.5% in 2014, affecting 422 million people [8], while depression is the most common mental health disorder and affects more than 264 million people worldwide [9]. Both are multifactorial, chronic conditions that require frequent reassessment and readjustment for their management [10].

The alarming rise of chronic conditions is increasingly straining health systems worldwide [11], and primary care has been widely proposed as one possible solution to tackle this issue [4,5], as the characteristics of chronic conditions make primary care the ideal level of care to manage and treat them. Here, we aim to explore how DMIA systems may be used specifically in the context of primary care, to enhance the consultations for diabetes and depression, as exemplars for chronic conditions. In order to do this, we delineated the particular characteristics of, and the tasks to be performed at, the primary care consultation that could be aided by a DMIA system. This helped identify the key topics a DMIA system should ask about before the primary care consultations for these chronic conditions. In addition, we discuss its potential benefits for the primary care consultation, as well as important considerations for the successful implementation of such a technology in the primary care context.

## Methods

A narrative review of the literature [12] was conducted between June and August 2019 and focused on two topics:

1. Characteristics of the primary care consultation in general (ie, scope, structure, communication, time, knowledge, and skills required) and on the primary care medical consultation for diabetes and depression specifically. A Google search was performed using the terms “primary care consultation” for obtaining general information regarding the primary care consultation from a variety of sources, including published work and grey literature. Additionally, a search through the Nanyang Technological University Library was performed using the same terms to locate textbooks regarding the general primary care consultation and for the consultation for diabetes and depression, specifically.
2. The impact of DMIA and CAHT systems on the medical consultation. Searches in PubMed and MEDLINE (Medical Literature Analysis and Retrieval System Online) were performed using the terms “computer-assisted history taking” and “automated history taking.”

A purposeful sample of articles was included based on relevance to build the Results and Discussion section. Robust evidence regarding CAHT and DMIA systems and their impact on the medical consultation was limited, as most studies evaluating these systems focused on other aspects, such as acquiring dietary or family history to estimate risk for diabetes, studies evaluating computer-assisted data collection for specific populations or related to screening, studies evaluating the test-retest reliability of computer-based medical histories, or efforts to generally computerize medicine, just to give some examples [13-18]. Relevant insights that were most useful for our purposes came mainly from commentaries, perspectives, and historical pieces [2,3,19], some of which analyzed or identified existing CAHT and DMIA software programs [1,20,21].

Thematic analysis of the extracted data from medical textbooks and literature helped identify higher-order themes and the specific, recurrent elements [22] that primary care consultations contain in relation to diabetes and depression. At the same time, we identified the specific areas where DMIA systems could improve the primary care consultation for these conditions. By combining both sources of information, we mapped the areas of the primary care consultation for diabetes and depression that might be improved by the use of DMIA systems. We did this for both the first presentation or consultation with the primary care provider and the follow-up visits, as the needs and tasks performed at these consultations are different. The information is reported following the structure of a narrative-style literature review [12].

Additionally, we developed a box (see Multimedia Appendix 1) representing the main identified tasks a primary care provider should perform at the initial (ie, screening and diagnosing) and follow-up primary care consultations (ie, general and clinical monitoring, checking for additional health issues, and coordinating); we completed it with the questions that a DMIA system should ask in order to facilitate these tasks, specifically for potential diabetes and depression patients. The box also includes the corresponding improvements for these primary care consultations.

## Results and Discussion

### Advantages and Disadvantages of DMIA Systems

No other medical consultation is as broad in terms of topics to be explored (ie, medical and clinical, psychological, and biosocial) [6,10,23] and limited in terms of time as the primary care consultation: mean consultation time is 5 minutes or less [24] or around 9 minutes, with general practitioners feeling that a consultation of less than 10 minutes for primary care was inadequate [25]. A DMIA system could improve primary care consultations in several ways, based on the benefits related to the medical consultation, broadly conceptualized. Textbox 1 and the following paragraphs summarize potential benefits and disadvantages that DMIA systems may bring forth for medical consultations in general, based on both theoretical work and on studies evaluating the capacities of these systems. In the textbox, some articles are perspective or commentary articles, theorizing about the capacities of DMIA systems [1-3,19,26], and others are studies evaluating systems and presenting results [21,27-29].

**Potential benefits and disadvantages of digital medical interview assistant (DMIA) systems for the medical consultation, compared to face-to-face consultations.**

Potential benefits of DMIA systems:

- Collect history for health screening or comprehensive clinical consultation [1]
- Collect more complete, accurate, and reliable information [1,3,19,26]
- Potential to increase diagnostic certainty [3]
- Considerably shorten the time spent on history taking, dictation, and documentation [1]
- Streamline office visit; allows for consultation to be focused on identified concerns and problems [3]
- Promote rapport, communication, and decision making [1,2,19]
- Can be integrated with electronic medical records, electronic health records, and online patient diaries, improving access to data [1,3,19,26]
- Enable triage prioritization and improve referral of patients [1]
- Help prepare patient and primary care provider for the consultation [3,19]
- Collect more sensitive information [1]

Potential disadvantages of DMIA systems:

- Technical issues—poor programming and design may result in [21]:
  Missing relevant information
  Collection of irrelevant information
  Erroneous information
- Human-computer interaction-related issues:
  Perceiving the computer interview as impersonal [1,27]
  Inability to detect nonverbal behavior [19]
- Duplication of effort—primary care provider attempts to confirm all responses [3]
- Require patient’s familiarity with technology and computer literacy [1,3,19]
- Require technical supervision and maintenance [1,19,28]
- Require a variety of factors (ie, legislative, organizational, and physician-level factors) to allow the successful implementation of eHealth innovations [28,29]

First, DMIA systems have the capacity to acquire a more comprehensive set of information than that attainable during a face-to-face consultation. This could provide greater insights into potential risk factors and, possibly, suggest a more accurate differential diagnosis, prior to seeing the patient [2,3,30]. Secondly, it allows for better use of face-to-face consultation time, as previous existing conditions and information about presenting complaints have already been identified before the patient enters the physician’s office [2,3]. This allows for a more streamlined consultation, where time that would have been spent on history taking is shifted to discussing management strategies, building rapport, and focusing on the relationship with the patient.

Third, it improves data quality [2,20,21], which in turn improves the diagnosis, as the quality of the diagnostic process is significantly affected by the accuracy of the information made available to the primary care provider. Fourth, DMIA systems include more up-to-date information, including that available from tests and other health care providers, which helps maintain continuity of information across different health care providers [30].

Additionally, once a diagnosis has been made by the primary care provider and subsequent consultations have been scheduled, DMIA systems can help flag specific aspects that need attention related to the ongoing management or treatment of the condition, and they can collect subsequent patient concerns prior to the follow-up consultation. As a result, the primary care provider in follow-up consultations can focus on guiding the patient regarding self-management aspects and provide further education that might be needed [3]. Alternatively, the information in a DMIA system may flag the need for specialist visits or management from other team members (ie, allied health professionals) in advance of seeing the patient, leading to earlier referrals or timely, urgent treatment, if needed [30].

However, there are also reported disadvantages of DMIA systems, which include possible technical issues, related to problems with programming or design, that may lead to missing or erroneous information [21]. Potential problems could occur as a result of a patient interacting with a computer, such as perceived impersonality of the health care provider–patient interaction or missing body language cues [1,19,27]. This last issue could be overcome by new facial and/or body movement recognition technologies, such as affective computing, where automated analysis of facial expressions can provide accurate depression diagnosis, for example [31]. Also, it has been described that some primary care providers attempt to confirm all the answers, duplicating efforts [3]. Finally, DMIA systems, as with other technologies, require technical supervision and maintenance [1,19,28], which may result in additional resource spending.

### Primary Care Consultation Tasks for Diabetes and Depression: Where Could DMIA Systems Make a Difference?

#### Prior to the Initial Consultation

##### Overview

Two important tasks of a primary care provider in the first consultation with a new patient are to identify a health condition, by case finding or screening, and diagnose that health condition [10,23,32-34]. The key aspect to these tasks is information gathering. As such, a preconsultation (ie, prior to the first consultation) DMIA system can exhaustively explore and ask questions about the different areas related to screening and diagnosis of a chronic condition prior to the first consultation, without particular time constraints.

##### Case Finding and Screening

Comprehensive case finding, along the lines of the biopsychosocial model, should include a generic set of questions exploring general health-related aspects, as well as more targeted screening questions, depending on the answers provided in the general questions section. General aspects may refer to lifestyle and health behaviors (ie, exercise, sleep duration, diet, general mood, alcohol and drug use, etc) and social behavior (eg, family, work conditions, and community aspects). Both of these question sets can provide hints for high-risk factors and behaviors, including patient beliefs and perspectives on the illness. Depending on the answers provided to the general screening questions, more targeted specific screening questions could be presented for the risk factors of hypertension, obesity, cancer, and other chronic conditions [35-37]. Answers from these could provide clinically relevant information for an accurate and earlier diagnosis (see Multimedia Appendix 1).

##### Diagnosing

Based on the responses from the case finding process, using branching logic, further questioning may dive deeper into specific risk factors to attempt a diagnosis. For example, for individuals at risk of diabetes, the system can ask questions related to possible prediabetes and signs and symptoms (see Multimedia Appendix 1) [35,36]; this may include plasma glucose level test results, if available [34]. For depression, the systems could focus on possible depressive and mood disorder diagnosis questions or screening tests, such as the 2-key question approach (see Multimedia Appendix 1) [37].

##### Urgent Care and Specialist Referral

The primary care provider, as the patient’s first contact and care coordinator, should have the ability to decide whether the patient needs emergency care or an urgent specialist referral [35-37]. Therefore, the primary care provider, aided by some of the responses provided by the patient prior to the consultation, could identify severe or uncontrolled cases of chronic conditions that require immediate and urgent attention. In his or her coordinating role, the primary care provider might promptly refer the patient to a specialist or to a hospital, based on the information provided and a quick face-to-face interaction, if needed.

#### Prior to the Follow-Up Consultation

##### Overview

Once a diagnosis has been reached, the primary care provider and patient would ideally establish a plan of action to treat and manage the condition. Key tasks of a primary care follow-up consultation include the control or monitoring of the condition [23,35-37], including identifying the potential occurrence of related additional health issues [35,36], as well as the coordination with relevant specialists regarding further workup [10,23]. A DMIA system can support these tasks by checking on the level of control and by monitoring the progress of the condition. It can explore additional health problems that may have arisen as a consequence of the condition and/or by adding additional useful information, such as information from other health care providers and/or lab tests, prior to the patient–health care provider follow-up consultation. Also, this can be done remotely and, hence, more frequently than the face-to-face follow-up consultation.

##### Control and Monitoring

A set of lifestyle and general questions (ie, exercise, sleep duration, diet, etc) will help check a patient’s compliance to lifestyle changes. For diabetes, these may evaluate changes in diet and exercise patterns, among others (see Multimedia Appendix 1). In the case of depression, the questions can relate to alcohol and drug use, sleeping patterns, and the situation at home and work, among others (see Multimedia Appendix 1).

The monitoring and control of chronic conditions highlight the importance of the patient becoming a partner in the management of their condition [36,38]. Relatedly, the system could also evaluate environmental and social conditions conducive to improved disease control. For example, in diabetes, a DMIA can explore accessibility to healthy food options at home and work and time available to exercise, among others. Regarding depression, cultural stigma regarding mental health and family support could be assessed [37].

After going through these sets of more general monitoring questions, the system can delve deeper into more clinically relevant questions, in order to monitor disease progression and check for medication adherence. It could ask questions about blood glucose levels (for diabetes), recurrent mood and depressed symptoms (for depression), and whether the patient has been taking medications properly.

##### Checking for Additional Health Issues

In addition to the chronic condition, the patient could have other associated health problems. The patient may not be aware that these other problems may be connected to the underlying chronic condition. In such cases, the DMIA system may be able to check for this prior to the follow-up consultation. In diabetes, it may assess for micro- or macrovascular complications (eg, kidney problems, peripheral vascular disease, foot ulcers, neuropathy, enteropathy, and ophthalmopathy) [35,36]. In depression, it can check for neurovegetative symptoms, difficulties concentrating, and suicidal ideations, among others [37,39].

##### Coordination

As mentioned above, given the role of coordinator that is taken on by the primary care provider, a DMIA system should ask questions related to the patient seeing other health care professionals, which may provide relevant additional information for the primary care provider. Often, when the patient is referred to specialists or other health care providers for additional treatment and tests, among other items, the information is not always transferred back to the primary care level [40]. Thus, these systems can check whether the consultations with other health care providers have been occurring and if they have been successful. For diabetes, this may relate to visits to the endocrinologist, ophthalmologist, nephrologist, among others [35,36]; for depression, this may relate to visits to the psychotherapist and psychiatrist [32,37,39]. Additionally, the systems may check with the patients whether there have been laboratory or other tests performed, and the primary care office could check with those external sites if the results have not made it back to the primary care level.

### Implications of Incorporating DMIA Systems Into the Primary Care Consultation

We described the role that a DMIA system could potentially play in managing consultations on diabetes and depression within the primary care context. The anticipated benefits of incorporating this technology into the primary care consultations for these chronic conditions should mirror those described in the literature: namely, a more comprehensive set of patient information items, better use of face-to-face consultation time, better-quality and more up-to-date data, and more frequent interactions with the patient, among others.

All the information gathered outside of the face-to-face consultation means that the actual time in consultation is better spent. Conversation can be streamlined to address the needs of the patient, such as building rapport, providing education, or responding to concerns, and to improve communication and patient understanding, which ultimately leads to increased patient satisfaction [41]. Relatedly, better-quality data prior to the consultation, as well as support for additional data coming from other levels of care or lab tests, allow for early detection of a chronic condition, earlier and enhanced treatment at the primary care level, better monitoring, and early management of possible complications, all of which translates into better care. In addition, as the DMIA communicates with the patient before and in between consultations, it improves the continuity of care and it gives the patient the feeling of being better cared for, as communication with the primary care provider via the DMIA system occurs more frequently.

Additionally, DMIA systems could improve the treatment and management of chronic conditions, as seen with diabetes and depression, without too much additional effort once the system has been set up. As mentioned above, chronic condition management usually follows a pre-established pattern. Therefore, it would not be difficult to develop a rules-based system to check case finding and diagnosis and appropriate management strategies. Then, once developed, the system could be deployed and repeated throughout, using the same branching logic and platform. Moreover, artificial intelligence could be leveraged and introduced into a DMIA system, which would potentially improve questioning algorithms and language, among other improvements.

### Important Implementation Considerations

For such a system to work well, there are several considerations that need to be taken into account. As presented earlier, there are some disadvantages that need to be bypassed or considered when implementing DMIA systems. Some patients may not be able to read the materials in digital form or may not be digitally literate; about 10% of the population chooses not to complete their histories on computers [3], although this figure may vary depending on how technologically acquainted people from different populations are. General practitioners sometimes try to confirm all the answers to the questions, thereby duplicating the work of the computer, which obviously impacts efficiency [3], although as technology and program design improve, there should be more confidence from these physicians in the results obtained by these programs. Additionally, there may be patient data entry and potentially added work for the primary care provider, as more issues might be highlighted and in need of attention. Moreover, bringing a DMIA system into everyday clinical practice requires interdisciplinary collaboration with a team of specialists and primary care physicians, patient advocates, computer scientists, big data analytics, and experience design [2].

Another consideration relates to the language to be used. The ideal DMIA system should provide a *human-like* interaction. For example, for a consultation ahead of the follow-up, it should phrase the questions in such a way that the patient feels as if they were continuing a previous conversation. Technologies today allow for human-like conversations in the form of a chatbot—also known as a machine conversation system, virtual agent, dialogue system, conversational user interface, and chatterbot—where a computer program interacts with users by using natural language precisely enough to simulate human conversation [42,43]. As such, the system may ask “Have you been able to adequately manage the [chronic condition] we discovered last week? Is there something you’d need extra help with?”

Another essential consideration relates to the factors that influence the adoption of eHealth innovations in health care in general, and primary care in particular. On the one hand, there are factors related to the adaptability, flexibility, and cost of the technology to be implemented [28]. DMIA systems need to be sufficiently flexible and affordable so that they easily enter and adapt to different primary care contexts and into already-existing working practices and systems. On the other hand, the environment in which the technology will be implemented needs to be ready for such a transformation and must include supportive policies, incentives, and leadership, as well as the appropriate resources for implementation [28,29]. In addition, a primary care provider’s knowledge and experience with digital technologies needs to be taken into account, as well as their willingness to incorporate a new technology into their everyday practice [28,29]. Only the proper alignment of these factors will enable a technology such as a DMIA system to be successfully implemented and, ultimately, improve care.

Finally, by transforming the primary care consultation in the way we describe here, a DMIA system would standardize the consultation and follow-up of chronic conditions and improve clinical care. We foresee that, given the described benefits, it should become the norm and a regular practice accompanying the primary care consultation if implemented in the appropriate way, by addressing the challenges and factors mentioned above. Moreover, it can become part of a suite of digital health technologies, amplifying its impact. For instance, by directly connecting a DMIA system to an electronic medical record (EMR) system and/or to a clinical decision support system (CDSS), it can have synergistic effects that may transform the way health care is provided in the future, such as providing rapid and remote access to care, improved triage, and more accurate diagnosis, just to name a few benefits. The answers provided to a DMIA system, which are automatically stored in a digital format, can populate whichever data fields are needed for an automated CDSS, providing support across a wide variety of clinical fields and issues [2,3,20]. In addition, clinical data can be standardized across all patients in contact with a health care system and that data can be included in a database to feed and support clinical and population health research [2].

### Conclusions

A DMIA system could enhance the primary care consultation and facilitate the management of diabetes and depression, and possibly other chronic conditions, which would hopefully make an impact in primary care. Earlier case finding and a more accurate diagnosis, due to more and better-quality data, paired with improved monitoring of disease progress, should improve the quality of care and keep the management of chronic conditions at the primary care level. A somewhat simple, easily scalable technology could go a long way to improve the health of the millions of people affected with chronic conditions, depending on the context and how successful its implementation is, especially if working in conjunction with already-established health technologies, such as EMRs and CDSSs.

## Appendix

Multimedia Appendix 1 Digital medical interview assistant questions for the cases of diabetes and depression.

## Abbreviations

CAHT: computer-assisted history taking
CDSS: clinical decision support system
DMIA: digital medical interview assistant
EMR: electronic medical record
MEDLINE: Medical Literature Analysis and Retrieval System Online
