# Supplementary material for: Improving the Primary Care Consultation for Diabetes and Depression Through Digital Medical Interview Assistant Systems: Narrative Review
Source: J Med Internet Res. 2020 Aug 28;22(8):e18109. doi: 10.2196/18109 (PMC7486669; doi:10.2196/18109)
Supplement: Multimedia Appendix 1 [file jmir_v22i8e18109_app1.docx]

Box: Digital medical interview assistant questions for the cases of diabetes and depression

| **PRE-FIRST/INITIAL CONSULTATION QUESTIONS** | | | |
| --- | --- | --- | --- |
| **Patient** | **All patients** | | **Improvements in primary care (PC) consultation** |
| General screening questions | How are your energy levels in these past 2 weeks? How well have you been sleeping these past 2 weeks? Have you been eating/drinking well? How often do you exercise? How many alcoholic drinks per week do you consume? | | - Earlier detection and diagnosis of chronic condition  - Improved use of consultation time (background and clinical information already filled out)  - More streamlined discussion to focus on flagged issues  - Opportunity for prompt referral if problems are out of the scope of PC or if there are urgent issues |
| **Answers/ results** | Sedentary, fatigue, excessive thirst 🡪 high risk diabetes | Trouble sleeping, poor appetite, high alcohol use 🡪 high risk mood disorder |  |
| **Patient** | **Potential diabetes patient** | **Potential mood disorder/depression patient** |  |
| Targeted screening questions | Do you have a history of: Family members with diabetes? Cardiovascular Disease? Hypertension? High cholesterol? Obesity? Gestational DM? Polycystic ovary syndrome? | Have you been experiencing changes in sleep, appetite, energy, concentration? Problems at home/work? Do you have a history of: psychosocial problems, past psychiatric conditions for you and/or family members? |  |
| **Answers/ results** | **Positive for high risk of diabetes** | **Positive for high risk of mood disorder** |  |
| Diagnosis questions | Have you had the following tests/results: Random plasma glucose concentration > 11.0 mmol/L, and/or fasting plasma glucose concentration >7.0 mmol/L, and/or oral failed glucose tolerance test | Have patient fill out a mini-mental state examination and/or ask “In the prior 2 weeks, have you felt down, depressed, or hopeless?” and “Have you noted a lack of interest or pleasure?” |  |
| **Answers/ results** | If positive**, possible diabetes diagnosis**  If dangerous**, urgent care referral** | If positive**, possible depression diagnosis**  If dangerous**, urgent care referral** |  |
| **PRE-FOLLOW-UP CONSULTATION QUESTIONS** | | | |
| **Patient** | **Diabetes** | **Depression** | **Improvements in PC consultation** |
| Control/ Monitoring general | Have you been eating healthy? Do you have healthy food options at home/work? How are your exercising patterns? Do you have available time/help at home to work out? Have you managed to lose weight? | How have you been feeling these past weeks? Have your energy/appetite/sleep levels improved? Have you managed to stop drinking alcohol? What does your family think of your condition? Have they been supportive/helpful? | - Better quality of data for improved monitoring  - Easier to flag problematic areas so that these can be discussed in the consultation  - Focus on education needed by the patient / review condition management strategies  - Improved capacity for referral, if needed  - Improved continuity and patient-doctor relationship |
| **Answers/ results** | **Adequate/problematic adaptation to lifestyle changes; adequate/problematic support at home/work to manage the condition 🡪 address issues accordingly** | **Adequate/problematic reestablishment of lifestyle/living conditions; adequate/ problematic support at home/work to manage the condition 🡪 address issues accordingly** |  |
| Control/ Monitoring clinical | What have been your blood glucose levels in the past week (key in values)? Have you had any hypo/hyper events? If so, how often and when? Have you been sticking to the Metformin schedule we planned? Have there been problems with any of the above? | Has your mood improved/been stable this week? Have you been feeling more/less depressed in the past days? Have you been sticking to the antidepressant therapy schedule we planned? Have there been any problems with any of the above? |  |
| **Answers/ results** | **Adequate/problematic management of diabetes 🡪 Focus on education for self-management, medication adherence strategies, additional support, etc.** | **Adequate/problematic progression of depression symptoms and/or medication management 🡪 Focus on issues that need attention** |  |
| Additional health issues | Have you been experiencing: lower back pain, difficulties urinating; pain in the legs, fatigue; foot problems, numbness/pain in hands or feet, stomach issues; vision problems? | Have you been experiencing additional and multiple health issues in various parts of your body? Have you lost interest in your usual sources of pleasure? Have you experienced suicidal thoughts or listened to voices in your head? |  |
| **Answers/ results** | **Identification of additional complications 🡪 treat and/or refer to a specialist accordingly** | **Identification of additional complications 🡪 treat and/or refer to a specialist accordingly** |  |
| Coordination questions | Were you able to see the podiatrist/ophthalmologist/ endocrinologist as we planned? Did they provide you with any information? Have you been referred for additional examinations or tests? Do you have and can you provide their results? | Have you been able to see the psychotherapist I recommended? How is that going? Have you been referred for additional examinations or tests? Do you have and can you provide their results? |  |
| **Answers/ results** | **- Relevant information from specialists/labs to be available for PC provider prior to the consultation**  **- If answers to any of the above indicate crisis, a referral process to specialist/hospital can be started. If control has been ok, management of patient can be kept at PC level** | |  |
